# Phytoplankton Community Structure Dynamics in Relation to Water Environmental Factors in Zhalong Wetland

**DOI:** 10.3390/ijerph192214996

**Published:** 2022-11-14

**Authors:** Xiaoyu Li, Yuxi Zhao, Fangying Chai, Hongxian Yu, Xu Sun, Di Liu

**Affiliations:** 1 School of Fishery, Zhejiang Ocean University, Zhoushan 316022, China; 2 College of Wildlife and Protected Area, Northeast Forestry University, Harbin 150040, China; 3 Northeast Institute of Geography and Agroecology, Chinese Academy of Sciences, Changchun 130102, China; 4 School of Management, Heilongjiang University of Science and Technology, Harbin 150020, China; 5 College of Fisheries and Life Science, Dalian Ocean University, Dalian 116023, China; 6 Heilongjiang Provincial Water Conservancy and Hydroelectric Power Investigation Design and Research Institute, Harbin 150080, China

**Keywords:** phytoplankton, water environmental factors, Zhalong wetland, canonical correspondence analysis (CCA), random forest

## Abstract

Phytoplankton, as the primary producer of the wetland water ecosystem’s food chain, are very sensitive to environmental changes. In order to explore the significance of phytoplankton in protecting ecosystem integrity, the wetland ecosystem in Zhalong wetland, one of the most important international wetlands, was selected as the research area. For the study, 81 sampling sites were set up in the whole wetland, and phytoplankton samples and water quality environmental factors were measured in spring, summer, and autumn of 2019. The phytoplankton community structure and water environmental factors were evaluated by canonical correspondence analysis (CCA). The main research findings are as follows: a total of 292 species and variants of phytoplankton belonging to 8 phyla and 110 genera were identified within Zhalong wetland in spring, summer, and autumn 2019. The total phytoplankton abundance and biomass in summer were higher than in spring and autumn, and *Cyclotella meneghiniana* was the most dominant species in three seasons and three areas. The results of random forest are generally consistent with the results of CCA in spring, when the main environmental factors affecting phytoplankton were NTU and WT; the result in summer and autumn agreed with those of CCA, which awaits further study. In addition, the phytoplankton is mainly affected by WT, depth, and DO in the lake area, TP, DO, and NTU in the river area, and WT in the wetland area.

## 1. Introduction

Phytoplankton, as primary producers in the wetland hydrosphere ecosystem, have rapid reproduction and are sensitive to environmental changes. The species composition abundance and the dominant species are important community structure characteristics and critical indicators reflecting water quality [1,2]. They are a critical component in determining the primary productivity of autotrophic water bodies and material cycling and energy flow [3]; for example, nutrient level, temperature, and light conditions have a strong influence on phytoplankton community structure [4,5,6], which is specifically manifested in their growth and reproduction rate. By controlling the intensity of photosynthesis and respiration of algae, the temperature directly affects the growth of algae, and the appropriate temperature for the growth of different algae varies. Some Cyanophyta are significantly correlated with total phosphorus, and their density is influenced by total phosphorus. Organic pollutants are oxidized and decomposed in water, and become nutrients that can be used by phytoplankton growth, thus affecting the number and distribution of phytoplankton [7,8,9]. In China’s wetlands, Bacillariophyta, Cyanophyta, and Chlorophyta account for a large proportion of phytoplankton community, which are greatly affected by seasonal changes and closely related to water temperature [10,11,12]. Changes in the other water environment factors will also lead to changes in community structure of phytoplankton [13]; therefore, the temporal and spatial distribution pattern of phytoplankton can reflect the changes in wetland ecological conditions. Evaluating the characteristics of phytoplankton community structure is one of the crucial ways to explore the functions of wetland ecosystems [2,14,15].

**Figure 1:**
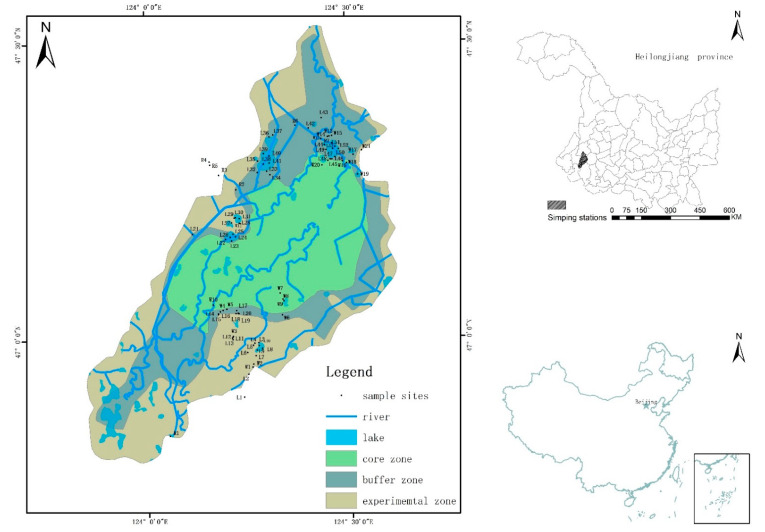
Sample sites within Zhalong National Nature Reserve.

Zhalong wetland in China was included on the List of Wetlands of International Importance in 1992. It is the largest National Nature Reserve dominated by cranes and other large waterfowl, and it is located on the left bank of Songnen Plain and in the marsh reed zone at the lower reaches of the Wuyuer and Shuangyang rivers. The study area (total area = 2100 km^2^) lies between 46°52′–47°32′ N and 123°47′–124°37′ E (Figure 1). This area has a temperate continental monsoon climate, with mean annual temperature from 2 to 4 °C. The multiyear average rainfall is 42 mm, mostly concentrated from July to September, accounting for more than 70% of total annual rainfall. A detailed understanding of the structure of the phytoplankton community in the wetland ecosystem can be of great use to understand the situation of the water environment and sustainable utilization of Zhalong wetland.

The primary focus of this research in Zhalong wetland was to explore the correlation between environmental factors and phytoplankton community structures. Random forest is a nonlinear model, which is widely used in agriculture, medicine, economics, satellite image processing, and other areas. With regard to phytoplankton research, random forest focuses on inversion and estimation, including the prediction of chlorophyll a and inversion of the smallest phytoplankton (<3 µm) [16,17]. However, the analysis of phytoplankton community structure rarely employs random forests. In order to evaluate the adaptability of the random forest model in studying the correlation of wetland phytoplankton and water environmental factors, canonical correspondence analysis (CCA) is used in the research of phytoplankton community structure; hence, it was applied to be the control analysis method [18]. These two methods (CCA and random forest) were used to clarify the spatial and temporal distribution regularities of environmental factors and phytoplankton community to provide primary data for discussing the succession pattern of the phytoplankton community structure within Zhalong wetland ecosystem. In addition, the environmental factors that limit the growth of phytoplankton are clarified, and the differences between the two methods are compared.

## 2. Materials

### 2.1. Sampling Sites

According to the regional climate and ecological environment characteristics of Zhalong wetland, phytoplankton, and environmental factors were collected in May (spring), August (summer), and October (autumn) 2019, and 81 sampling sites were set up in lake, wetland, and river. The sampling sites in the three seasons were the same: lake area (L1~L53), where natural lakes are full of aquatic vegetation and aquaculture; wetland area (W1~W21), where the dominant vegetation group of Zhalong wetland is reed and cattail; river area (R1~R7), where the sites are mainly distributed in Wenghai drain, and there is substantial high overlap with human-disturbed environments.

First, three duplicate quantitative samples of phytoplankton (1 L) were collected according to the following parameters: (a) at a water depth below 3 m, the sample was collected from 0.5 below the surface; (b) at a water depth of 3–10 m, mixed samples were taken at the surface and bottom layers; (c) at a water depth more than 10 m, mixed samples were taken at the surface and middle and bottom layers. Collection at each site was conducted using a phytoplankton net (20 cm diameter, 50 cm length, 64 μm mesh), with horizontal hauls in the subsurface (0.5 m depth). Each 1 L phytoplankton sample was preserved in Lugol (1%) and formaldehyde (5%) for later identification and enumeration. A light microscope (MOTIC BA410) was used for applied to obtain the species and quantity of phytoplankton (V = 0.1 mL), which were identified to the lowest taxonomic level using keys by described in [19,20].

Seven environmental factors were recorded using a multiparameter probe (YSI6600) in the field: water temperature (WT), chloride ion (Cl^−^), pH, ammonium nitrogen (NH_4_^+^-N), conductivity (EC), turbidity (NTU), and dissolved oxygen (DO). Water transparency (SD) was measured using a Secchi disc. Water samples were taken at a depth of 0.5 m below the surface, stored in 1 L PE bottles, and analyzed for total nitrogen (TN), total phosphorus (TP), and chemical oxygen demand (COD_cr_); the method referred to water and wastewater monitoring method IV [21].

### 2.2. Data Processing

#### 2.2.1. Analysis of Species Dominance

$$D=\frac{n_{i}}{N}.f_{i},$$

where *n_i_* is the individual number of organisms of a species at one sample site, *N* is the total individual amount, and *f_i_* is the frequency of occurrence of species *i* in all samples. A species is considered as dominant if *D* ≥ 0.02.

#### 2.2.2. One-Way ANOVA

The significance of the differences of environmental parameters in three seasons was analyzed using one-way ANOVA. Data were analyzed using SPSS 21.0 software.

#### 2.2.3. For Canonical Correspondence Analysis

The abundance and continuous environmental variables (except pH) of selected phytoplankton species were normalized using the formula lg(1 + x) [22,23] to make the data more normally distributed. Canoco software (version 5.0) ( Jan Leps and Petr Smilauer, Bohemia, Czech) was used for detrended correspondence analysis (DCA). According to the results of DCA, if the greatest length of the gradient value of the four axes is more than 3.0, canonical correspondence analysis (CCA) should be selected; if it is between 2.0 and 3.0, both CCA and redundancy analysis (RDA) can be selected; if it is less than 2.0, RDA should be chosen. The greatest lengths of gradient values of spring, summer, and autumn were between 2.0 and 3.0; thus, CCA was selected. CCA is a type of direct gradient analysis that links environmental variables with community composition, so that the variables can explain the community composition. In this research, we assessed how the phytoplankton community varied along with the environmental factors.

CCA is an ordination analysis that connects environmental variables and community composition so that the variables can be used to interpret the composition pattern of the community structure at study sites [24,25], and evaluate how the community structure varies along with environmental factors. In the CCA plot, species are represented by points and arrows show environmental variables. The direction of the arrow represents the directions in the plot in which the particular variables increase, and the length of arrows indicates importance. These points and arrows together reflect the distribution of species with each environmental variable; points close to the arrow of an environment, indicate a tendency toward significant results when the environment changes. Therefore, a smaller included angle between the axis and environmental factors in the diagram indicates a higher correlation.

#### 2.2.4. Random Forest Method

The random forest (RF) model is capable of handling nonlinear data well and has significant advantages in characterizing interactions between variables. Furthermore, it is insensitive to unbalanced and missing data and has import efficiency in processing with regression and classification. RF method was implemented in the randomForest package in R. The R software package, a port of Breiman and Cutler’s [26] original Fortran code by Liaw and Wiener, version 4.6–6 [27], was used. The increase in the mean squared residual (%IncMSE) is usually used to measure the importance of the variable (water environmental factor) [28]. If one environmental factor is more important, the error value predicted by the RF model will increase after it is randomly replaced with other arbitrary environmental factors. Therefore, a higher %IncMSE indicates a more critical variable (environmental factor). The environmental factors are ranked according to %IncMSE values, and the top-ranked factors are strongly correlated with phytoplankton growth. The evaluation of RF predictive performance of the model through cross-validation and partialPlot showed the relationships between environmental factors and phytoplankton abundance and indicated a marginal effect, i.e., a particular environmental effect of phytoplankton abundance while ignoring all other environmental factors.

## 3. Results

### 3.1. Spatiotemporal Distribution Characteristics of Phytoplankton Community within Zhalong Wetland

During the investigation period, a total of 292 species within Zhalong wetland belonging to 110 genera were detected among the samples, including Cyanophyta (19 species), Bacillariophyta (103 species), Chrysophyta (nine species), Xanthophyta (one species), Pyrrophyta (six species), Cryptophyta (four species), Euglenophyta (31 species), and Chlorophyta (119 species).

In the whole region, the dominant species were Chlorophyta, Bacillariophyta, and Euglenophyta, comprising 86.64% of the total. Among them, Chlorophyta accounted for the largest proportion with 40.75%, followed by Bacillariophyta with 35.27%, Euglenophyta with 10.62%, Cyanophyta with 6.51%, Chrysophyta with 3.08%, Pyrrophyta with 2.05%, Cryptophyta with 1.37%, and Xanthophyta with 0.34%.

There are more phytoplankton species in spring (May) than in summer (August) and autumn (October). In autumn, low water temperature becomes the limiting factor for the growth of phytoplankton rather than nutrients. Affected by the flood disaster in 2019, many rivers in Heilongjiang Province exceeded an alert level in summer. As a result, the water level of rivers and lakes within Zhalong wetland rose significantly, and the turbidity of water bodies also increased greatly, resulting in fewer phytoplankton species. Overall, there were fewer species in Pyrrophyta and Chrysophyta, mainly Glenodinium and Dinobryon, for which weak acidic conditions with less organic matter content and excellent water quality are suitable for growth.

In the entire Zhalong wetland, 138 species of phytoplankton belonging to seven phyla and 66 genera were found at sampling points in the river area, including Cyanophyta (four genera, four species), Bacillariophyta (25 genera, 68 species), Chrysophyta (three genera, three species), Pyrrophyta (one genus, one species), Cryptophyta (three genera, three species), Euglenophyta (three genera, 10 species), and Chlorophyta (27 genera, 49 species). Of the 138 phytoplankton species, 86 species were present in spring, along with 71 in summer, and 62 in autumn.

A total of 181 species of phytoplankton, belonging to eight phyla and 82 genera were found at sampling points in the wetland area, including Cyanophyta (seven genera, 15 species), Bacillariophyta (21 genera, 60 species), Chrysophyta (three genera, five species), Pyrrophyta (two genera, three species), Cryptophyta (three genera, three species), Euglenophyta (five genera, 15 species), Chlorophyta (40 genera, 79 species), and Xanthophyta (one genus, one species). Of the 181 phytoplankton species, 97 species were present in spring, along with 122 in summer and 113 in autumn.

A total of 245 species of phytoplankton, belonging to eight phyla and 103 genera were found at sampling points in the lake area, including Cyanophyta (nine genera, 17 species), Bacillariophyta (26 genera, 80 species), Chrysophyta (three genera, four species), Pyrrophyta (three genera, five species), Cryptophyta (three genera, four species), Euglenophyta (six genera, 24 species), Chlorophyta (52 genera, 110 species), and Xanthophyta (one genus, one species). Of the 245 phytoplankton species, 181 species were present in spring, along with 176 in summer and 140 in autumn.

### 3.2. Spatiotemporal Variation of Phytoplankton Abundance and Biomass within Zhalong Wetland

**Figure 2:**
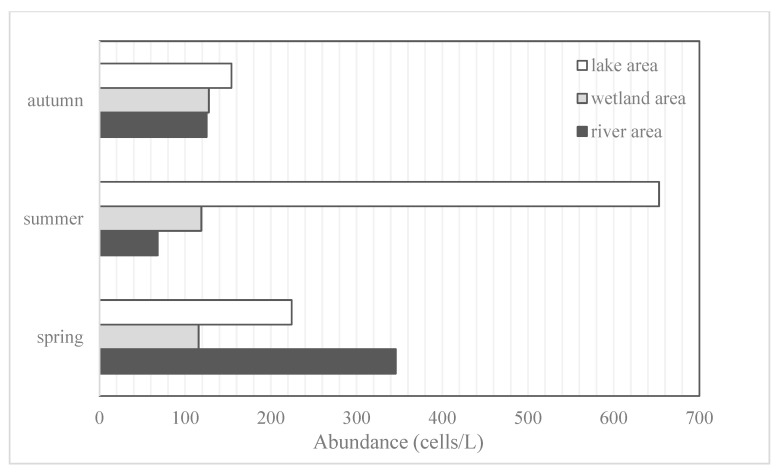
Distribution of average phytoplankton abundance in sampling area.

The abundance of phytoplankton at each sampling point ranged from 38.4 × 10^4^ to 6560.4 × 10^4^ cells/L, and the average abundance at each sampling point in the river, wetland, and lake areas is shown in Figure 2. The highest abundance of phytoplankton was found in summer, followed by spring, with the lowest in autumn; the average abundance was the highest in summer. On the other hand, the average abundance of the wetland area did not change significantly during the three seasons.

The total abundance of phytoplankton within Zhalong wetland was 66,036.4 × 10^4^ cells/L; the largest share was Chlorophyta (60.23%), followed by Bacillariophyta (27.05%), Cryptophyta (3.60%), Euglenophyta (3.54%), Cyanophyta (3.15%), Chrysophyta (1.96%), Pyrrophyta (0.46%), and Xanthophyta (0.01%).

In spring, the abundance of *Cyclotella meneghiniana* was the highest (2425.2 × 10^4^ cells/L), followed by *Synedra acus* (2085.6 × 10^4^ cells/L); in summer, the abundance of *Chlorella vulgaris* was the highest (26,174.4 × 10^4^ cells/L), followed by *Cyclotella meneghiniana* (1764 × 10^4^ cells/L); in autumn, the abundance of *Cyclotella meneghiniana* was the highest (1956 × 10^4^ cells/L), followed by *Chlorella vulgaris* (1516.8 × 10^4^ cells/L). The latter two are widely distributed species and have always been the dominant species in the Zhalong wetland [29]. Under suitable environmental conditions, the increase rate for *Cyclotella meneghiniana* can be high. It is more than helpful for it to become the competitive species in natural water bodies, as it can still reproduce slowly at low temperatures (about 10 °C). *Chlorella vulgaris* mostly grows in small fertile water bodies and is common in lakes, ponds, and shallow harbors.

#### 3.2.1. Phytoplankton Biomass

**Figure 3:**
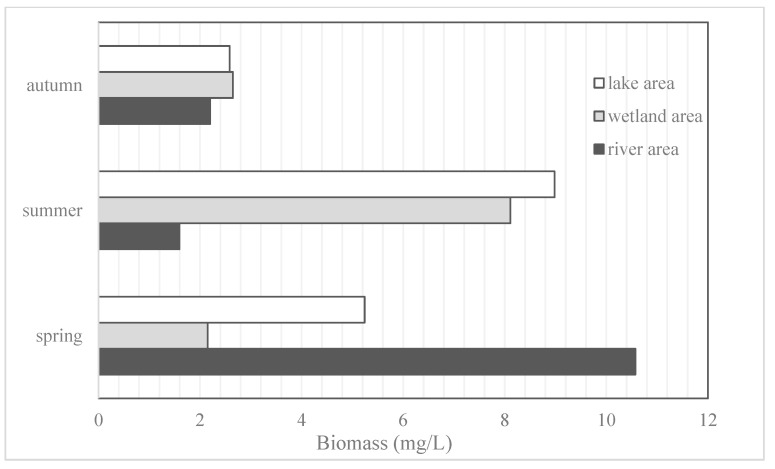
Distribution of average phytoplankton biomass in sampling area.

The phytoplankton biomass varied between 0.70 and 107.49 mg/L at the sampling sites; the average biomass of each sampling site in the river, wetland, and lake areas is shown in Figure 3. Generally, phytoplankton biomass is the highest in summer and the lowest in autumn. The highest average biomass appeared in the river area in spring. The total biomass in the three seasons was 1262.52 mg/L, with Cyanophyta accounting for the largest proportion (36.31%).

#### 3.2.2. Temporal and Spatial Distribution Characteristics of Dominant Species of Phytoplankton within Zhalong Wetland

Table 1 shows the dominance and distribution of phytoplankton species. The dominant species overlapped and changed in the three seasons: three species in Bacillariophyta: *Synedra acus*, *Cyclotella meneghiniana*, and *Ceratoneis Closterium*, two in Chlorophyta: *Ankistrodesmus angustus* and *Chlorella vulgaris*, and one in Cryptophyta: *Rhodomonas lacustris*.

There were four dominant species in lake areas, with dominance between 0.024 and 0.418, seven dominant species in river areas, with dominance between 0.020 and 0.285, and nine dominant species in wetland areas, with dominance between 0.031 and 0.120. In comparison, Sanjiang Plain (Sanhuanpao wetland, a similar high-latitude natural environment to Zhalong Wetland) [30,31], is dominated by Bacillariophyta, Chlorophyta, and Cyanophyta in spring, summer, and autumn, whereas no significant response of Cyanophyta occurred in Zhalong wetland. The dominant phytoplankton species in the Baiyangdian wetland in North China were similar to those in the Zhalong wetland (Chlorophyta and Bacillariophyta), and the dominant autotroph was Chlorophyta of the genus Chlorella [32]. It was observed that spatial heterogeneity and seasonal change led to a difference in dominant phytoplankton species. Different aspects of wetland habitats (climate, temperature, vegetation, hydrology, etc.) caused differences in the distribution of dominant phytoplankton communities.

### 3.3. Temporal and Spatial Distribution Characteristics of Water Environmental Factors within Zhalong Wetland

A total of 12 environmental factors were evaluated in the survey (Table 2). One-way ANOVA revealed that these seasonal differences in these factors were significant (* *p* < 0.05) except water transparency (SD) (*p* > 0.05).

It can be observed from Table 3 that some differences in the spatial distribution of environmental factors, including TN, COD_cr_, Cl^−^ and NH_4_^+^-N changed significantly. Due to the purification effect of submerged vegetation and emergent vegetation on water quality, the average value of TN in wetland areas was the lowest, and, in lake areas with high nutrients, the average value of total nitrogen was high. The mean value of COD_cr_ was the lowest in lake areas and the highest in river areas; WT was not significant in spatial variation. The average value of Cl^−^ was the highest in lake areas. There are many lakes within the Zhalong wetland, most of them stocked with fish, which would lead to a high Cl^−^ content.

### 3.4. CCA of Phytoplankton Community Structure and Environmental Factors within the Zhalong Wetland

The results of one-way ANOVA showed an SD *p*-value > 0.05 (0.762); hence, they were not included in the environmental factors for CCA. The environmental factors selected for CCA were TN, TP, COD_cr_, WT, Cl^−^, pH, NH_4_^+^-N, EC, NTU, DO, and depth, and the chosen phytoplankton were species with an occurrence frequency of ≥10% during the sampling period, i.e., 54 phytoplankton species (Appendix A). They were analyzed from two aspects: seasons (spring, summer, and autumn) and study area (lake, river, and wetland).

#### 3.4.1. CCA of Phytoplankton in Spring, Summer, and Autumn

**Figure 4:**
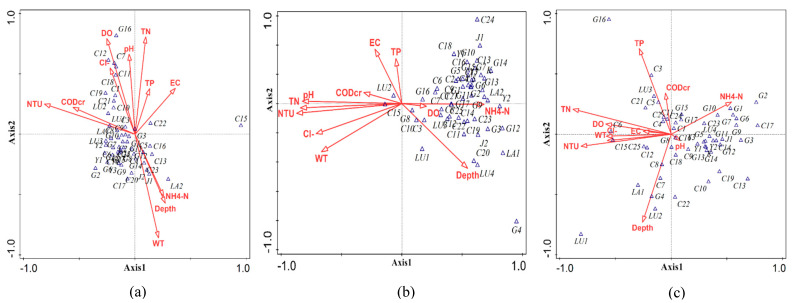
CCA between phytoplankton species and environmental factors in (**a**) spring, (**b**) summer, and (**c**) autumn.

The results of this analysis showed that the 11 environmental factors explained 40.3%, 30.4%, and 33.0% of the phytoplankton species in the spring, summer, and autumn, respectively. The length of the environmental vector is proportional to the correlation between the environmental factor and the phytoplankton ordination (Figure 4).

The ordination plot of the species–environment relationship revealed the following: (1) NTU, TN, and WT had a significant influence on the growth of phytoplankton in spring. The species are projected on the extension line of the environment vector; the species closer to the arrow of the environment vector were the most competitive when the environment changed significantly, and vice versa. Several phytoplankton species appear on the environment vector arrows in spring, indicating that fewer species were tolerant to a single environmental factor. (2) The phytoplankton showed a significant relationship with NTU in summer. The rainy season is in summer, and the influx of a large amount of sediment makes the water turbid and interferes with the growth of phytoplankton; therefore, NTU is inversely proportional to phytoplankton. NH_4_^+^-N and water depth are proportional to phytoplankton. The majority of phytoplankton species in summer appear on the NH_4_^+^-N vector arrow, indicating that NH_4_^+^-N is also an important environmental factor for phytoplankton in summer. (3) TN and TP had remarkable effects on phytoplankton in autumn. NH_4_^+^-N is also an important environmental factor affecting the growth of phytoplankton in autumn.

#### 3.4.2. CCA of Phytoplankton in Lake, River, and Wetland Areas

**Figure 5:**
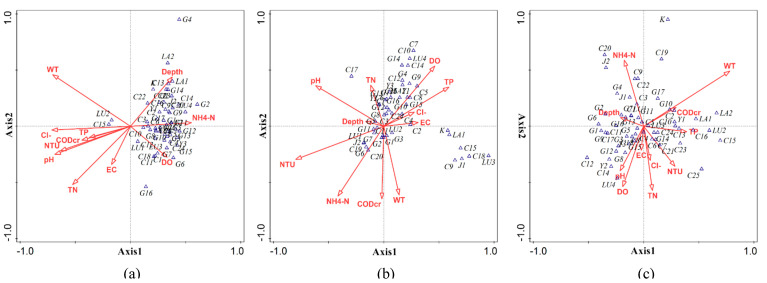
CCA between phytoplankton species and environmental factors: (**a**) lake area; (**b**) river area; (**c**) wetland area.

The CCA of phytoplankton in lake, river, and wetland areas showed that 11 environmental factors explained 29.1%, 63.6%, and 31.0%, respectively, of the total variance of species distribution. The biplots (Figure 5) indicate a strong relationship between phytoplankton species and environmental variables. (1) There is a strong correlation between phytoplankton and WT and TN in lake areas. The seasonal variation of water temperature is evident. The abundance of phytoplankton in lake areas is in the order summer > spring > autumn. In summer with higher temperatures, thermophilic species of green algae such as Chlorella vulgaris were dominant. Some lakes within Zhalong wetland are blind lakes and have no river recharge; thus, the nutrient content high, which promotes the reproduction and growth of phytoplankton. (2) NTU is an important environmental factor affecting phytoplankton in summer. The sampling sites in the river area were mainly concentrated in Wenghai drain with large sediment content, which limited the light, resulting in a decline in the phytoplankton growth rate and individual density. (3) For wetland areas, WT is an essential environmental index. Wetland is an area of open water with lush vegetation and swamps; as it is an ecological ecotone with a complex habitat, the species structure of phytoplankton is diverse. Therefore, WT is an environmental factor with insignificant spatial heterogeneity in wetlands, and it plays a more significant role in wetland areas.

### 3.5. Analysis of the Influence of Water Environmental Factors on Phytoplankton Abundance Using the Random Forest Method

#### 3.5.1. Random Forest Analysis of Phytoplankton and Environmental Factors in Spring

**Figure 6:**
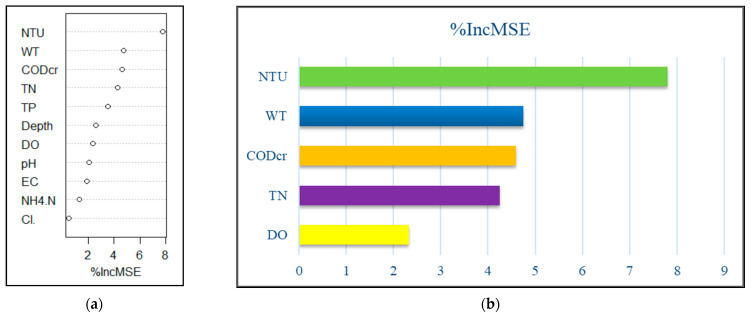
Environmental factors ranked in descending order of importance based on (**a**) %IncMSE and (**b**) major environmental drivers of phytoplankton abundance in spring.

**Figure 7:**
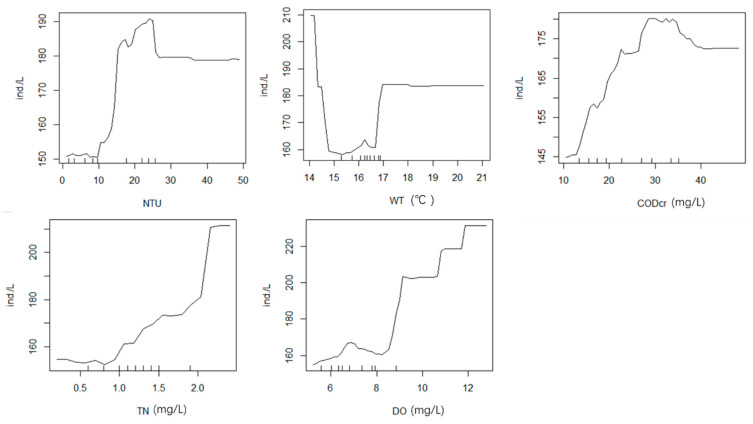
Effects of five environmental drivers (NTU, WT, COD_cr_, TN, and DO) on phytoplankton in spring.

The random forest model with 11 environmental factors (TN, TP, COD_cr_, WT, Cl^−^, pH, NH_4_^+^-N, EC, NTU, DO, and depth) explained 26.22% of the total variance. Figure 6a presents the results of random forest analysis, showing the environmental variables ranked by importance. All models were trained and tested using tenfold cross validation, and the results (Figure 6b) revealed NTU (%IncMSE = 7.800), WT (%IncMSE = 4.753), COD_cr_ (%IncMSE = 4.594), TN (%IncMSE = 4.244), and DO (%IncMSE = 2.329) as the important variables influencing phytoplankton in spring; these five factors explained 29.86% of the total variance, which was higher than the previous value (26.22%). These results are generally consistent with those of the phytoplankton CCA in spring. The relationship between environmental factors and phytoplankton is not a simple linear relationship. A nonlinear model (random forests) can better handle such cases. The line chart of the correlation between five main environmental factors and phytoplankton based on random forest (Figure 7) shows that phytoplankton abundance increased with higher concentrations of TN and DO. This indicates that increased TN and DO is conducive to abundant growth of phytoplankton in a certain range. WT in spring was low and did not exceed 17 °C; the line chart shows that, when WT was higher than 17 °C, the trend became a straight line, resulting in the water quality being clear in spring. At NTU values more than 30, the line in the figure became a straight line, indicating that the impact on phytoplankton reached the limit.

#### 3.5.2. Random Forest Analysis of Phytoplankton and Environmental Factors in Summer

**Figure 8:**
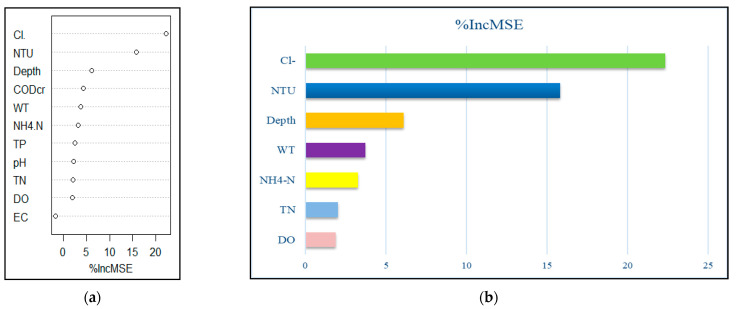
Environmental factors ranked in descending order of importance based on (**a**)%IncMSE. and (**b**) major environmental drivers of phytoplankton abundance in summer.

The 11 environmental factors explained 52.19% of total variance in summer, and the input feature importance ranking is shown in Figure 8a. Feature importance from the random forest cross-validation results (Figure 8b) showed Cl^−^ (%IncMSE = 22.33), NTU (%IncMSE = 15.79), depth (%IncMSE = 6.090), WT(%IncMSE= 3.725), NH_4_^+^ -N (%IncMSE = 3.245), TN (%IncMSE = 2.024), and DO (%IncMSE = 1.870) as the important variables influencing phytoplankton in summer, explaining 53.63% of the total variance, which was higher than the previous value (52.19%). There was a deviation in the random forest result from that of CCA in summer.

**Figure 9:**
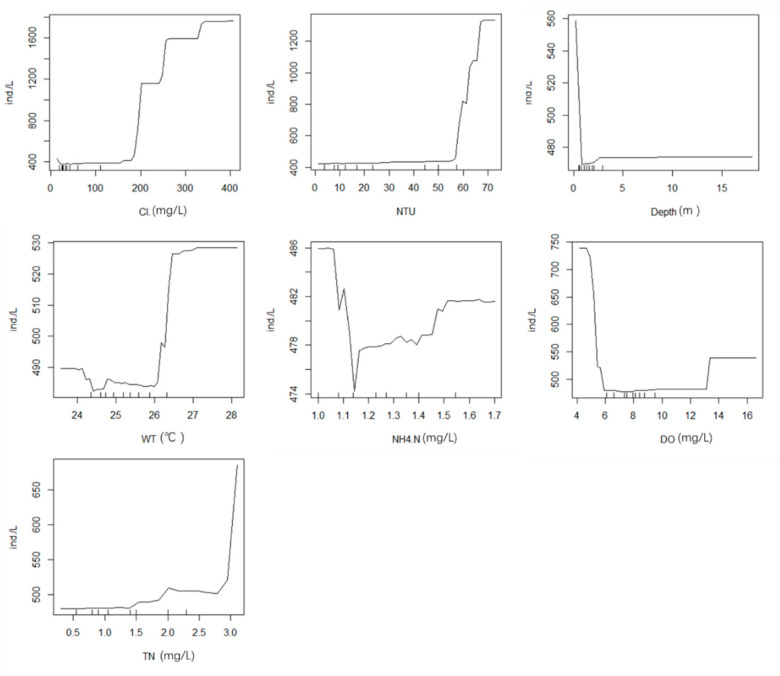
Effects of seven environmental drivers (Cl^−^, depth, NTU, DO, NH_4_^+^-N, TN, and WT) on phytoplankton in summer.

A line chart of the correlation between the seven main environmental factors and phytoplankton in summer (Figure 9) shows that, when the value of Cl^−^ was less than 200 mg/L, it had a minor effect on species abundance, but when Cl^−^ concentration was more than 200 mg/L, the phytoplankton abundance growth appeared stepwise on the line; the abundance increased rapidly when NTU > 60, TN > 3 mg/L, and WT > 26 °C, and there was a negative correlation between phytoplankton abundance and water depth.

#### 3.5.3. Random Forest Analysis of Phytoplankton and Environmental Factors in Autumn

**Figure 10:**
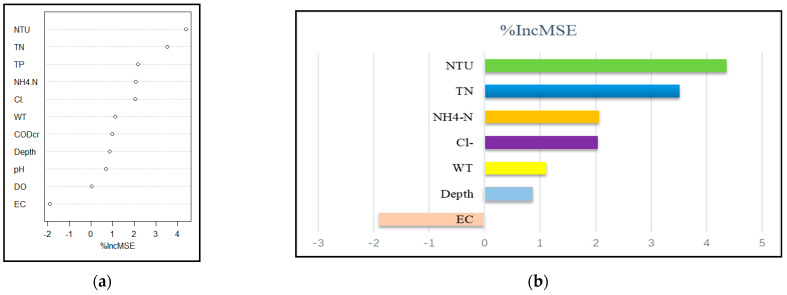
Environmental factors ranked in descending order of importance based on (**a**) %IncMSE and (**b**) major environmental drivers of phytoplankton abundance in autumn.

The 11 environmental factors explained 25.10% of the total variance in autumn, and the input feature importance ranking is shown in Figure 10a. After cross-validation, seven main environmental factors were found to affect phytoplankton abundance in autumn (Figure 10b): NTU (%IncMSE = 4.365), TN (%IncMSE = 3.517), NH_4_^+^ -N (%IncMSE = 2.064), Cl^−^ (%IncMSE = 2.040), WT (%IncMSE = 1.112), depth (%IncMSE = 0.852), and EC (%IncMSE = −1.903), explaining 29.05% of total variance, which is higher than previous value (25.10%).

**Figure 11:**
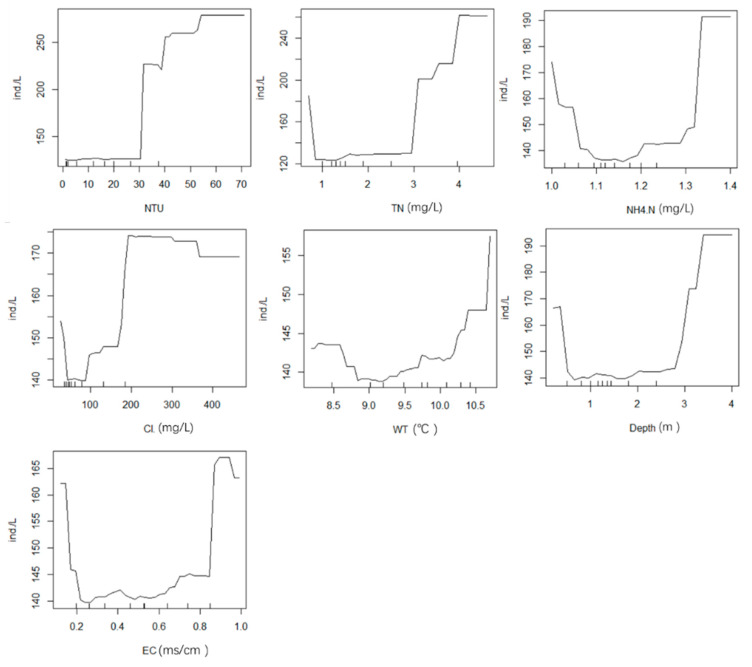
Effects of seven environmental drivers (NTU, TN, NH_4_^+^-N, Cl^−^, WT, depth, and EC) on phytoplankton in autumn.

As shown on the line chart for autumn (Figure 11), the phytoplankton abundance increased with turbidity and with NTU. There was no significant correlation between other environmental factors and phytoplankton.

#### 3.5.4. Random Forest Analysis of Phytoplankton and Environmental Factors in Lake, River, and Wetland Areas

The random forest model for phytoplankton and environmental factors appeared to have a poor fit to the data in each study area. After cross-validation, the total percentage of variance explained by the random forest model with environmental factors decreased from 16.65% to 10.27% for lake areas, and from 38.7% to 21.37% for river areas, while a negative value (−14.86%) was obtained for wetland areas. It was observed that the random forest model was not applicable to correlation analysis between phytoplankton and environmental factors within the Zhalong wetland area. This may be due to the overfitting of the model to the details of the noise in the dataset. That is, the training and test sets showed tremendous variability in the dataset. However, the specific mechanism needs to be studied further.

## 4. Discussion

In a temperate climate, Chlorophyta is dominant at high nutrient levels in early spring and summer, while Bacillariophyta is dominant at low temperatures. Therefore, within Zhalong wetland, the largest numbers of Chlorophyta were found in spring and summer 2019 (41.95% and 45.73%), and the largest numbers of Bacillariophyta were found in autumn (39.63%). Nutrients are generally considered to be the most important driving factors for the growth of phytoplankton. They mutually affect and transform each other [33,34]. With high temperature and high TN concentration (the highest average value was in the lake area) co-action in the summer, the average abundance of phytoplankton was much higher at each sampling point in the lake area than in the river and wetland areas. In spring, there was higher overall abundance of *Cyclotella meneghiniana* and *Synedra acus* (2425.2 × 10^4^ cells/L and 2085.6 × 10^4^ cells/L respectively), with larger individuals; consequently, the highest average biomass was observed in the river area. Thermophilic species increased rapidly in the lake area in summer, such as *Chlorella vulgaris*, which was absolutely dominant in abundance (26,174.4 × 10^4^ cells/L), but the individuals were so small that they did not dominate in biomass. Zhalong wetland has four distinct seasons and prominent climate characteristics; Zhalong Wetland has a diverse, variable ecosystem with a variety of phytoplankton species. With reference to the previous studies in the Zhalong wetlands, as well as the results of this thesis, abundance and biomass of phytoplankton varied significantly with seasons [2,35]. The CCA of phytoplankton and environmental factors in Zhalong wetland showed that multiple environmental factors correlated with the phytoplankton community structure. In CCA for spring, NTU, TN, and WT were the main environmental factors affecting phytoplankton structures, among which the species of Bacillariophyta were directly proportional to WT. In the mid-latitude semi-humid and semi-arid region, where Zhalong Wetland is located, there are normal seasonal fluctuations in water temperature, with a low–high–low tendency in spring, summer, and autumn. Phytoplankton biomass and abundance also vary seasonally with changes in water temperature. The results of CCA for summer showed that NTU was the main environmental factor affecting phytoplankton, with a negative correlation. In addition, NH_4_^+^-N was an essential restrictive environmental factor. According to the results of CCA for autumn, TN and TP were the main environmental factors limiting the growth of phytoplankton. CCA indicated that the main environmental factors affecting phytoplankton abundance were WT, TN, and NTU in lake, river, and wetland areas, respectively. Compared to river areas and wetland areas, several lakes in the Zhalong Wetland have much higher amounts of TP and TN, and these lakes are small, confined water bodies. Additionally, the water body is seriously eutrophicated due to a lack of emergent aquatic plants, a small surface area, and weak self-purification capabilities. As a result, the phytoplankton community structure in the lake region is greatly impacted by NH_4_^+^-N. In summary, WT, nitrogen, and phosphorus nutrients were the most critical environmental factors for phytoplankton in Zhalong wetland.

Before the implementation of ecological water replenishment in Zhalong wetland, except for annual precipitation, there was almost no external water supply; the Wuyuer and Shuangyang rivers were the primary water sources. The construction of agricultural small ponds and reservoirs upstream intercepted two-thirds of the water of Wuyuer River and almost all the water of Shuangyang River, thus blocking the flow into Zhalong wetland. After introducing the Nenjiang River as a water source for ecological water replenishment, the sluice is opened in June every year, which alleviates the problem of water shortage in Zhalong wetland to a certain extent. In the rainy season, rainwater and flood peaks caused by opening the gate lead to domestic sewage and farmland fertilizer being washed into the wetland, increasing the nitrogen and phosphorus nutrients in the region’s water, which is a main advantage for phytoplankton in Chlorophyta.

In this paper, using the random forest model to study the critical water environmental factors affecting the growth of phytoplankton, the results for spring are consistent with those of CCA, whereas the results for summer showed a deviation between the two. In the random forest analysis, Cl^−^ was the most important environmental factor affecting phytoplankton, not NTU, but it was the second most important environmental factor in CCA. There were also some deviations between the random forest method and CCA results for autumn, although total TN was equally important in both methods. In studying the correlation between phytoplankton abundance and environmental factors, the partialPlot diagram showed that (1) increased of TN and DO in spring would promote an increase in phytoplankton abundance; (2) in summer, NTU, TN, and WT had no significant effect on increasing phytoplankton abundance within a specific range; however, but when the values exceeded a specific range, abundance increased rapidly, showing a correlation with water depth; (3) in autumn, phytoplankton abundance increased with increasing NTU. Nevertheless, in evaluating the correlation between phytoplankton and environmental factors in each study area (lake, river, and wetland), compared with CCA, the random forest model revealed its lack of adaptability.

Research on phytoplankton in Zhalong wetland is focused on the influence of different environmental gradients on phytoplankton biomass, diversity, and community structure distribution [29,36]. Two methods, canonical correspondence analysis (CCA) and random forest model, have advantages and disadvantages in studying the influence of water environmental factors on phytoplankton. The major advantage of CCA is that it can simultaneously display the results of sampling points, species, and environmental factors in CCA ordination; a disadvantage is that rare species need to be eliminated to overcome the “arch effect”. PartialPlot in the random forest model solves the problem that the importance index can not reflect the positive and negative correlation; the effects of relevant variables on phytoplankton abundance can be observed in a partialPlot diagram. The defect of partialPlot is that it is numerically similar to the marginal effect of a linear model; it seems to reflect the relationship between a dependent variable and an independent variable after ignoring other independent variables. However, when this relationship is obvious, its fragmented fitting distorts the actual connection between the two objects. The random forest method outperforms CCA in the following ways: (1) First, apart from characterizing positive and negative correlation with environmental factors, partialPlot can also clearly show the trend of correlations within a specific numerical range so that the results can be analyzed and explained according to the practical significance of water ecology; (2) second, the random forest method is not sensitive to missing original data, and it does not need to eliminate rare species to ensure the integrity of the original data, as CCA does.

## 5. Conclusions

In this study, the phytoplankton population in the Zhalong wetland was dominated by Chlorophyta, with a total proportion of 40.75%, followed by Bacillariophyta, with 35.27%. The dominant phytoplankton species varied from Bacillariophyta to Cyanophyta and Chlorophyta depending on spatiotemporal variation in Zhalong wetland. We selected 54 species of phytoplankton (occurrence frequency ≥ 10%) combined with water environmental factors that showed significant differences in each season after one-way ANOVA to analyze the influence of water environmental factors on the phytoplankton community. CCA results showed that NTU, TN, WT, and TP had a remarkable effect on phytoplankton within Zhalong wetland. The results of random forest regarding the main environmental factors affecting phytoplankton in spring showed good agreement with the results of CCA. The top two environmental factors are turbidity (NTU) and water temperature (WT); however, the results of random forest deviated from those of CCA in summer and autumn. In conclusion, the most important environmental parameters for phytoplankton in Zhalong Wetland were WT, nitrogen, and phosphorus nutrients. Among them, TP and TN were probably the main control environmental factors to further eutrophication problems. Our findings suggest that managers of the Zhalong wetland should optimize the upstream water allocation, enhance the internal ecology of the wetland, and preserve its function as the “Earth’s kidney”. 

## Figures and Tables

**Table 1:** Distribution of dominant phytoplankton species within Zhalong National Nature Reserve.

	Species	Dominant Index					
		Spring	Summer	Autumn	Lake Area	River Area	Wetland Area
Bacillariophyta	*Synedra acus*	0.116		0.112	0.048	0.165	0.117
Bacillariophyta	*Synedra tabulata*					0.099	
Bacillariophyta	*Cyclotella meneghiniana*	0.132	0.042	0.163	0.076	0.285	0.120
Bacillariophyta	*Fragilaria brevistriata*					0.025	0.038
Bacillariophyta	*Fragilaria capucina*					0.020	
Bacillariophyta	*Ceratoneis closterium*	0.021					
Chlorophyta	*Ankistrodesmus angustus*	0.027		0.052	0.024	0.026	0.033
Chlorophyta	*Chlorella vulgaris*		0.310	0.067	0.418		0.045
Chlorophyta	*Chlamydomonas globosa*						0.031
Cryptophyta	*Rhodomonas lacustris*			0.021			0.046
Cryptophyta	*Cryptomonas ovata*					0.028	0.074
Chrysophyta	*Dinobryon divergens*						0.034

**Table 2:** Mean values of environmental factors in Zhalong wetland in three seasons (±standard error).

Environmental Factors	Spring	Summer	Autumn	*p*-Value
TN (mg/L)	1.22 ± 0.05	1.22 ± 0.07	1.83 ± 0.11	0.000 **
TP (mg/L)	0.10 ± 0.01	0.20 ± 0.01	0.20 ± 0.01	0.000 **
COD_cr_ (mg/L)	24.07 ± 1.02	27.10 ± 0.54	24.99 ± 0.75	0.023 *
WT (°C)	16.27 ± 0.10	25.20 ± 0.09	9.60 ± 0.08	0.000 **
Cl^−^ (mg/L)	35.19 ± 2.54	68.34 ± 10.97	101.14 ± 11.68	0.000 **
pH	9.43 ± 0.03	9.36 ± 0.05	9.66 ± 0.05	0.000 **
SD (cm)	58.12 ± 5.18	59.60 ± 5.44	63.37 ± 4.92	0.762
NH_4_^+^-N (mg/L)	1.51 ± 0.03	1.24 ± 0.02	1.13 ± 0.01	0.000 **
EC (ms/cm)	0.41 ± 0.03	0.46 ± 0.03	0.51 ± 0.03	0.029 *
NTU	15.20 ± 1.61	26.66 ± 2.58	17.35 ± 2.30	0.001 **
DO (mg/L)	7.15 ± 0.17	7.84 ± 0.18	9.91 ± 0.22	0.000 **
Depth (m)	1.13 ± 0.07	1.94 ± 0.33	1.31 ± 0.08	0.011 *

* p < 0.05; ** *p* < 0.01.

**Table 3:** Spatial distribution of mean values(±SE) of environmental factors within Zhalong wetland.

	Spring			Summer			Autumn		
Environmental	Lake Area	River Area	Wetland Area	Lake Area	River Area	Wetland Area	Lake Area	River Area	Wetland Area
Factors									
TN	1.31 ± 0.07	1.23 ± 0.10	0.99 ± 0.08	1.25 ± 0.09	1.10 ± 0.27	1.17 ± 0.13	1.85 ± 0.15	1.84 ± 0.19	1.77 ± 0.16
TP	0.11 ± 0.01	0.08 ± 0.01	0.09 ± 0.01	0.17 ± 0.01	0.26 ± 0.03	0.27 ± 0.01	0.17 ± 0.01	0.23 ± 0.04	0.25 ± 0.02
CODcr	20.61 ± 0.96	32.30 ± 2.04	30.04 ± 2.26	26.56 ± 0.61	28.57 ± 2.58	28.37 ± 1.16	24.29 ± 0.94	29.40 ± 1.67	25.27 ± 1.48
WT	16.08 ± 0.12	16.57 ± 0.13	16.67 ± 0.24	25.46 ± 0.11	25.14 ± 0.20	24.58 ± 0.08	9.66 ± 0.10	9.45 ± 0.22	9.50 ± 0.15
Cl^−^	38.52 ± 3.48	30.38 ± 8.19	28.40 ± 3.05	80.19 ± 16.32	30.75 ± 6.26	46.78 ± 7.00	116.06 ± 16.96	43.81 ± 6.28	82.60 ± 10.64
pH	9.47 ± 0.03	9.55 ± 0.07	9.29 ± 0.07	9.52 ± 0.05	9.14 ± 0.13	8.99 ± 0.07	9.76 ± 0.07	9.49 ± 0.11	9.44 ± 0.07
NH_4_^+^-N	1.52 ± 0.03	1.45 ± 0.06	1.49 ± 0.10	1.20 ± 0.02	1.31 ± 0.09	1.33 ± 0.05	1.11 ± 0.01	1.20 ± 0.03	1.18 ± 0.02
EC	0.44 ± 0.03	0.37 ± 0.09	0.34 ± 0.05	0.41 ± 0.03	0.61 ± 0.10	0.53 ± 0.06	0.51 ± 0.03	0.37 ± 0.07	0.57 ± 0.06
NTU	16.94 ± 2.29	20.51 ± 3.20	9.04 ± 1.41	31.32 ± 3.37	19.86 ± 8.52	14.22 ± 3.55	21.16 ± 3.34	9.64 ± 2.61	10.32 ± 1.51
DO	7.48 ± 0.23	6.32 ± 0.31	6.59 ± 0.17	8.13 ± 0.24	7.59 ± 0.44	7.18 ± 0.21	10.04 ± 0.26	8.62 ± 0.41	10.01 ± 0.52
Depth	1.21 ± 0.10	1.16 ± 0.17	0.90 ± 0.09	2.36 ± 0.49	1.10 ± 0.27	1.18 ± 0.13	1.21 ± 0.10	1.54 ± 0.19	1.50 ± 0.16

## Data Availability

The datasets analyzed during the current study are available from the corresponding author on reasonable request.

## Appendix A

**Table A1:** List of phytoplankton with occurrence frequency ≥10% in spring, summer, and autumn in Zhalong Wetland.

Phylum	Species	Code	Phylum	Species	Code
Cyanophyta	*Anabaena azotica*	LA1	Euglenophyta	*Euglena pisciformis*	LU4
Cyanophyta	*Leptolynbya* sp.	LA2	Pyrrophyta	*Glenodinium* sp.	K
Bacillariophyta	*Fragilaria brevistriata*	G1	Chlorophyta	*Chlamydomonas ovalis*	C1
Bacillariophyta	*Fragilaria capucina*	G2	Chlorophyta	*Chlamydomonas globosa*	C2
Bacillariophyta	*Fragilaria virescens*	G3	Chlorophyta	*Chloromonas* sp.	C3
Bacillariophyta	*Melosira granulata var.angustissima*	G4	Chlorophyta	*Ankistrodesmus angustus*	C4
Bacillariophyta	*Synedra acus*	G5	Chlorophyta	*Ankistrodesmus acicularis*	C5
Bacillariophyta	*Synedra tabulata*	G6	Chlorophyta	*Tetrastrum staurogeniae*	C6
Bacillariophyta	*Synedra ulna*	G7	Chlorophyta	*Scenedesmus dimorphus*	C7
Bacillariophyta	*Cyclotella meneghiniana*	G8	Chlorophyta	*Scenedesmus quadricauda*	C8
Bacillariophyta	*Navicula radiosa*	G9	Chlorophyta	*Scenedesmus bijuga*	C9
Bacillariophyta	*Navicula cincta*	G10	Chlorophyta	*Pediastrum boryanum*	C10
Bacillariophyta	*Navicula exigua*	G11	Chlorophyta	*Kirchneriella lunaris*	C11
Bacillariophyta	*Navicula pupula*	G12	Chlorophyta	*Crucigenia tetrapedia*	C12
Bacillariophyta	*Navicula cryptocephata*	G13	Chlorophyta	*Staurastrum* sp.	C13
Bacillariophyta	*Cymbella affinis*	G14	Chlorophyta	*Cosmarium depressum*	C14
Bacillariophyta	*Gyrosigma acuminatum*	G15	Chlorophyta	*Chlorella vulgaris*	C15
Bacillariophyta	*Ceratoneis closterium*	G16	Chlorophyta	*Westella botryoides*	C16
Bacillariophyta	*Cocconeis placentula*	G17	Chlorophyta	*Mougeotia* sp.	C17
Chrysophyta	*Dinobryon divergens*	J1	Chlorophyta	*Oocystis elliptica*	C18
Chrysophyta	*Kephyrion boreale*	J2	Chlorophyta	*Oocystis lacustris*	C19
Cryptophyta	*Cryptomonas ovata*	Y1	Chlorophyta	*Haematococcus pluvialis*	C20
Cryptophyta	*Chroomonas caudata*	Y2	Chlorophyta	*Tetraedron minimum*	C21
Cryptophyta	*Rhodomonas lacustris*	Y3	Chlorophyta	*Tetraedron trilobulatum*	C22
Euglenophyta	*Euglena oxyuris*	LU1	Chlorophyta	*Tetraedron trigonum*	C23
Euglenophyta	*Euglena caudata*	LU2	Chlorophyta	*Golenkinia radiata*	C24
Euglenophyta	*Euglena acus*	LU3	Chlorophyta	*Tetrachlorella alternans*	C25
